# The native stem holoparasitic *Cuscuta japonica* suppresses the invasive plant *Ambrosia trifida* and related mechanisms in different light conditions in northeast China

**DOI:** 10.3389/fpls.2022.904326

**Published:** 2022-09-23

**Authors:** Wei-Bin Wang, Fan-Fan Gao, Wei-Wei Feng, Qi-Ye Wu, Yu-Long Feng

**Affiliations:** ^1^ College of Plant Protection, Shenyang Agricultural University, Shenyang, China; ^2^ Liaoning Key Laboratory for Biological Invasions and Global Changes, Shenyang Agricultural University, Shenyang, China; ^3^ College of Bioscience and Biotechnology, Shenyang Agricultural University, Shenyang, China

**Keywords:** *Ambrosia trifida*, plant invasions, *Cuscuta japonica*, carbon, nitrogen, growth, reproduction, stable isotope composition

## Abstract

Increasing evidence from low-latitude ranges has demonstrated that native parasitic plants are promising biocontrol agents for some major invasive weeds. However, related mechanisms and the effect of environments on the control effect of the parasite are still unclear. In addition, few related studies have been conducted in high latitude (>40°), where the exotic plant richness is the highest in the globe, but natural enemies are relatively scarce. During field surveys, a *Cuscuta* species was found on the cosmopolitan invasive weed *Ambrosia trifida* L. in Shenyang, northeast China. Here, we first studied the impacts of the parasite on the invader at three sites with different light regimes and related mechanisms, then the haustorial connections between the parasite and the invader using anatomy and measurement of carbon (C) and nitrogen (N) stable isotope compositions (δ^13^C, δ^15^N), and finally identified the parasite using two molecular marks. The parasite was identified as *C. japonica* Choisy. This native holoparasitic vine posed serious C rather than N limitation to the invader, explaining its greatly inhibitory effects on the invader. Its negative effects were stronger on reproductive relative to vegetative growth, and at high relative to low light habitats, which indicated that the higher the vigor of the host is, the higher the impact of the parasite pose. The parasite could establish haustorial connections with phloem, xylem, and pith of the invader and thus obtain resources from both leaves and roots, which was confirmed by difference of δ^13^C and δ^15^N between the two species. The parasite had significantly higher leaf C concentrations and δ^13^C than its invasive host, being a strong C sink of the parasitic association. Our results indicate that *C. japonica* may be a promising biological control agent for the noxious invader in China.

## Introduction

Currently, biological invasion is widely considered as one of the major drivers of global biodiversity loss (Díaz et al., 2019). As a major group of invasive alien species (IAS) worldwide (Pyšek et al., 2008), range-expanding exotic plants can override, displace local species, and dominate invaded communities (Hejda et al., 2009; Zheng et al., 2015; Iqbal et al., 2021). The enemy release hypothesis (ERH) and biotic resistance hypothesis (BRH) are two long-standing hypotheses explaining the success of some alien plants (Qin et al., 2013; Zheng et al., 2015; Zhao et al., 2020). The former posits that introduced plants experience fewer natural enemies in their new relative to old ranges and can therefore proliferate more vigorously (Keane and Crawley, 2002). The seeds and leaves of *Ambrosia trifida* L. (target plant in current study) suffer much less attack from aboveground enemies in its invasive range China than in the USA, its native range (Zhao et al., 2020). In addition, belowground enemies also strongly inhibit seed germination and seedling growth of the invader in the USA. BRH holds that interactions with native species limit invaders’ impacts. Native parasitic plants restrict invasive hosts and seem more environment-benign than introduced enemies, which may pose non-target effects in their new ranges besides host shift (Van Driesche et al., 2010). These native parasitic plants, which keep their invasive hosts in check, present a sustainable element of IAS management schemes (Yu et al., 2011; Cirocco et al., 2016; Wang et al., 2020; Těšitel et al., 2021). However, the relevant evidence is still limited.

Parasitic plants tend to forage whichever species is fast-growing and abundant in habitats, two of the major features of invasive plants. High growth rates can increase the competitive ability of plants at the expense of defense, showing a ‘growth-defense trade-off’ (Herms and Mattson, 1992; Feng et al., 2009). The invasive plants with high vigor were hypothesized to be the preferred hosts of local parasites (Alpert, 2006; Callaway and Maron, 2006). Studies have demonstrated that phototropic *Cuscuta* spp. prefer foraging hosts with high resources (Kelly, 1992; Li et al., 2007), plant height, and growth rates. These traits generally exist in many cosmopolitan invasive plant species, particularly the first (Liu et al., 2022). In China, a higher leaf nitrogen (N) concentration and less structural defense (toughness) have been found in 47 invasive plants compared with their co-occurring natives (Huang et al., 2020), including two notorious invasive plants, *A. trifida* and *A. artemisiifolia* L. These susceptible invaders with high abundance have more chances of being infected. Host preference has been found on many invasive plant species in their dominated communities (Pennings and Callaway, 1996; Prider et al., 2009; Yu et al., 2011).

Evidence is growing that native parasitic plants effectively constrain invasive hosts and favor community restoration. For instance, the native root hemiparasitic *Rhinanthus* has been demonstrated to extensively suppress the growth of native problematic *Calamagrostis epigejos* (L.) Roth and used as an effective tool for restoring the grasslands invaded by *C. epigejos* in Czech Republic (Těšitel et al., 2017; Těšitel et al., 2018). In South Australia, another native hemiparasitic vine (*Cassytha pubescens* R. Br. (Lauraceae)) decreased 40%–60% of biomass of the invasive shrub *Ulex europaeus* L., while it had no effects on two native hosts in a series of greenhouse experiments, regardless of light or N conditions (Cirocco et al., 2016; Cirocco et al., 2017). In addition, Cirocco et al. (2018) found that *C. pubescens* efficiently impaired the physiological performance of *U. eurolaeus* across all three sites in the fields. In South China, the native *Cuscuta australis* (R. Br.) greatly decreased the plant cover (by 50%–60%) and rooted node number (by 29%–64%) of three clonal perennial invaders and promoted the restoration of invaded communities (Yu et al., 2011). To date, however, related studies chiefly focused on the parasite impacts on perennial invaders, and little progress has been made in the novel biocontrol scheme for annual invasive hosts (especially in high latitudes), particularly in their reproduction, which determines their future spreads.

The isotopic signature of carbon (C) and N provided much detailed information on nutrient retention and theft in host–parasite associations in a long-term scale. Fractionation of isotopes indicated that most reactions discriminate against heavy isotopes and result in a relatively lower isotopic signature than the original source. The isotopic composition of leaf N (δ^15^N) provided a time-integrated measure of the external source, biological sink, and allocation of N over the period when the leaf material is metabolized (Kalcsits et al., 2014), and leaf C isotopic signature (δ^13^C) represented long-term leaf-level intrinsic water-use efficiency and degree of stomatal control over photosynthesis (Farquhar et al., 1989). A growing number of studies take advantage of the isotopic fractionation of C to reveal the C sources and effects of hemiparasites on their native or alien perennial hosts (Scalon and Wright, 2015; Cirocco et al., 2016; Cirocco et al., 2018). However, no study has addressed the specific N ‘trade agreement’ between parasites and their alien hosts.

*Ambrosia trifida* L. (giant ragweed; Asteraceae) is a summer annual herb native to temperate North America (Bassett and Terasmae, 1962), including Canada, the United States, and northern Mexico. This plant is currently a cosmopolitan invasive weed in temperate Eurasia where it easily overgrows most of the native vegetation and dominates communities. In both its native and introduced ranges, *A. trifida* is widely known as a noxious weed (Allard, 1943; Zhao et al., 2020). The tall height of the invader magnifies dispersal of its anemochorous pollens, which are productive and allergic to people who suffer from hay fever, and confers competitive advantage over resident vegetation by forming greater cover, as reported in other invaders (Feng et al., 2007). In China, it was first recorded in Tieling in the 1930s, and then in Shenyang, Liaoning, northeast China, in the early 1950s (Guan et al., 1983; Feng, 2020). It can grow up to 4.3 m in height in moist, fertile, and disturbed habitats, such as the banks of agricultural drainage ditches and roadsides. It has spread into more than 17 provinces, ranging from northeast Heilongjiang to southwest Sichuan and been listed in the first batch of national priority for IAS management (MAG, 2013). Thus, novel and efficient biocontrols are strongly needed for *A. trifida*.

In the summer of 2018, a previously unreported *Cuscuta* species was first observed on an *A. trifida* population growing along the north bank of Hunhe River in Shenyang, Liaoning. This novel host–parasite association was also found along Shenshui East Road, Shenyang, in 2019 and 2020. In this study, we first determined the negative effects of the parasite on the invader at three sites with different light intensities and the underlying mechanism then tested their haustorial connections using the anatomy and measurement of C and N isotopic signatures and finally identified the parasite using two molecular marks. We hypothesized that this parasite may efficiently restrict the growth and reproduction, by causing strong resource limitation in *A. trifida*. To test the hypothesis, we measured a suite of plant traits from leaf morphology, stem growth, to reproduction, such as leaf size, stem height, and achene production. We also evaluated and interpreted the resource budget of the association in terms of N and C. Our study may contribute to understanding the mechanism underlying the impacts of holoparasites on invasive plants, providing a basis for controlling invasive weeds using holoparasites in high-latitude areas.

## Materials and methods

### Study sites

Our study was carried out in Shenhe District, Shenyang City, Liaoning Province, northeast China. Shenyang is a semi-humid continental monsoon climate. Here, the annual mean temperature is 8.525°C, and the annual mean precipitation is 698.5 mm from 1981 to 2010. The accumulative rainfall and mean temperature in July, August, and September of 2020, during which our study was conducted, were 55.7, 333.5, and 82.8 mm, and 25.2, 24.5, and 18.0°C, respectively (data from National Meteorological Information Center of China).

Three sites were selected along Shenshui East Road (41°48′57.33″N, 123°33′06.19″E; 57 m a.s.l). The parasite on *A. trifida* in this area was first found in 2019. It may come from the nearby bank of Hunhe River, where the parasite on the invader was first found in 2018. Site 1 was on the slope of the road, with full sunshine; site 2 on the shoulder of the road, under the canopy of tall trees (*Ulmus pumila* L.); and site 3 on the road margin, under the canopy gap. The impacts of this parasite on *A. trifida* were determined in late August, 2020, when the invader was in the vigorous reproductive growth stage.

### Samplings and measurements

At each site, six pairs of *A. trifida* plants infected and -uninfected by the parasite were selected, and the two plants in each pair were spaced within 1 m apart in order to decrease the potential effects of habitat heterogeneity. The main stem of each sampled plant had 16–17 nodes to ensure that the plants were at a similar growth stage. For each *A. trifida* plant sampled, height was measured using a stainless steel rule, and the ratio of infected main stem length to plant height as a measure of parasite infection load; the stem diameter at 40-cm height (base of stem usually had an irregular circle or oval shape) was measured using a reading Vernier caliper, and the number of branches (≥5 cm), male super-inflorescences, and all young achenes were counted, respectively.

Relative chlorophyll content was measured on the fourth leaf (from tip) of each plant using a SPAD 502 Plus Chlorophyll Meter (Minolta, Osaka, Japan). Then, the leaf was collected and placed in a Ziplock bag and immediately taken back in an ice box to our laboratory to measure the leaf area with a LI-3000C portable leaf area meter (LICOR, Lincoln, NE, USA). The leaves were oven-dried separately to a constant weight at 60°C and weighted using an analytical balance. Leaf mass per area (LMA) was calculated as the ratio of leaf biomass to area. To decrease the potential effects of the possible difference in leaf thickness on the relative chlorophyll content, the SPAD value measured for each leaf in the field was converted to mass-based SPAD value: the product of the measured SPAD value and LMA.

The dried leaves were ground using a ball mill (GT200, Grinder, Beijing, China) and stored separately in Ziplock bags for measuring C and N concentrations and their stable isotope composition (δ^13^C and δ^15^N). The measurements were performed using an elemental analyzer (Elementar Vario Micro Cube; Elementar, Langenselbold, Germany) connected to an isotope ratio mass spectrometer (IsoPrime100; IsoPrime, Ltd., Cheadle, UK) at Shenyang Institute of Applied Ecology, Chinese Academy of Science, Shenyang, China.

Stems of the parasite on the third internode of each sampled *A*. *trifida* plant were carefully collected for measurement of C and N concentrations and their stable isotope compositions. The methods were the same as those for leaf materials.

For testing the haustorial connection between the invader and the parasite, i.e., the parasitism between the two species, ≈ 2-cm-long stem segments of the host penetrated by the *Cuscuta* stems were collected, rinsed with distilled water, and fixed with the formalin-aceto-alcohol (FAA) fixing fluid. Anatomy of the haustorium was examined using a paraffin section (Fischer et al., 2008), and micrographs were taken on a light microscope (Nikon Eclipse Ci-L, Tokyo, Japan) with a digital camera (Nikon DS-U3).

### Identification of the parasite

The stems of the parasite on *A. trifida* were collected at four sites (see **Supplementary Table 1**), specimen of the parasite–host association (SYAUB24418) was collected from the north bank of Hunhe River, and deposited in the Herbarium of Shenyang Agricultural University. Total genomic DNA of these stems was extracted using the Plant Genomic DNA Kit (Tiangen Biotech, Beijing, China). The sequences for the nuclear rDNA internal transcribed spacer 2 (ITS2) and chloroplast *trn*L intron were obtained using the novel primers S2F (5′-ATGCGATACTTGGTGTGAAT-3′)/S3R (5′-GACGCTTCTCCAGACTACAAT-3′) (Chen et al., 2010), and universal primers cB49317F (5′-CGAAATCGGTAGACGCTACG-3′)/dA49855R (5′-GGGGATAGAGGGACTTGAAC-3′) (Taberlet et al., 1991), respectively. The parasite DNA template (570 ng, 2 μl) and 0.5 μl of each primer (10 μM) were added to 12.5 μl 2× San *Taq* ^®^ Fast PCR mix buffer (Sangon Biotech, Shanghai, China), and sterile ddH_2_O was added to a final volume of 25 μl. The PCRs of ITS2 and *trn*L ran the same program as follows: initial denaturation at 94°C for 4 min, followed by 35 cycles of denaturation at 94°C for 10 s, annealing at 55°C for 20 s, extension at 72°C for 66 s, and a final extension for 5 min. These amplifications were performed in a T100 thermocycler (Bio-Rad, Hercules, CA, USA). The amplicons were cleaned with a Sanprep Column PCR Product Purification Kit (Sangon Biotech), cloned into the T1 vector with a p-*Easy* ^®^-T1 Simple Cloning Kit (Transgene Biotech, Beijing China), and sequenced at Sangon Biotech.

The GenBank accession numbers for these sequences are as follows: for ITS2 (435 bp): MK070163, MW486637, MW486627, and MW486639; for *trn*L (386 bp): MK879389, MW618675, MW618665, and MW618676. Phylogenetic trees of these sequences of ITS2 and *trn*L were generated using the Bayesian inference method in MrBayes 3.2.7a (Ronquist et al., 2012). The best-fit model was determined by jModelTest 2.1.10. Reference sequences that are highly similar to the ones from our study (13 rITSs >99.1%, 7 *trn*Ls *>*99.48%) were retrieved from GenBank (**Supplementary Table 2**).

### Statistical analyses

Two-way ANOVA was used to test the effects of the parasite treatments or the groups of species and parasitic treatments (parasite, and *A. trifida* with and without infection; only for N and C concentrations, C:N ratio, δ^13^C and δ^15^N), study sites, and their interaction on all variables measured in this study; one-way ANOVA was used to test the difference in each variables among sites for *A. trifida* with the same parasitic treatments or the parasite. For N- and C-related variables, the difference among the parasite and *A. trifida* with and without infection at each site as well as the mean values of three sites was tested using one-way ANOVA; for the remaining variables, independent sample *t*-test was used to analyze the difference between *A. trifida* with and without infection at each site as well as the mean values of three sites. The numbers of branch and male super-inflorescences were log_3_-transformed; LMA and achene number were square-root-transformed in order to meet the conditions of variance analysis. All analyses were done using SPSS 13.0 (Chicago, IL, USA).

## Results

### Inhibitory effects of the parasite on the invader

**Figure 1:**
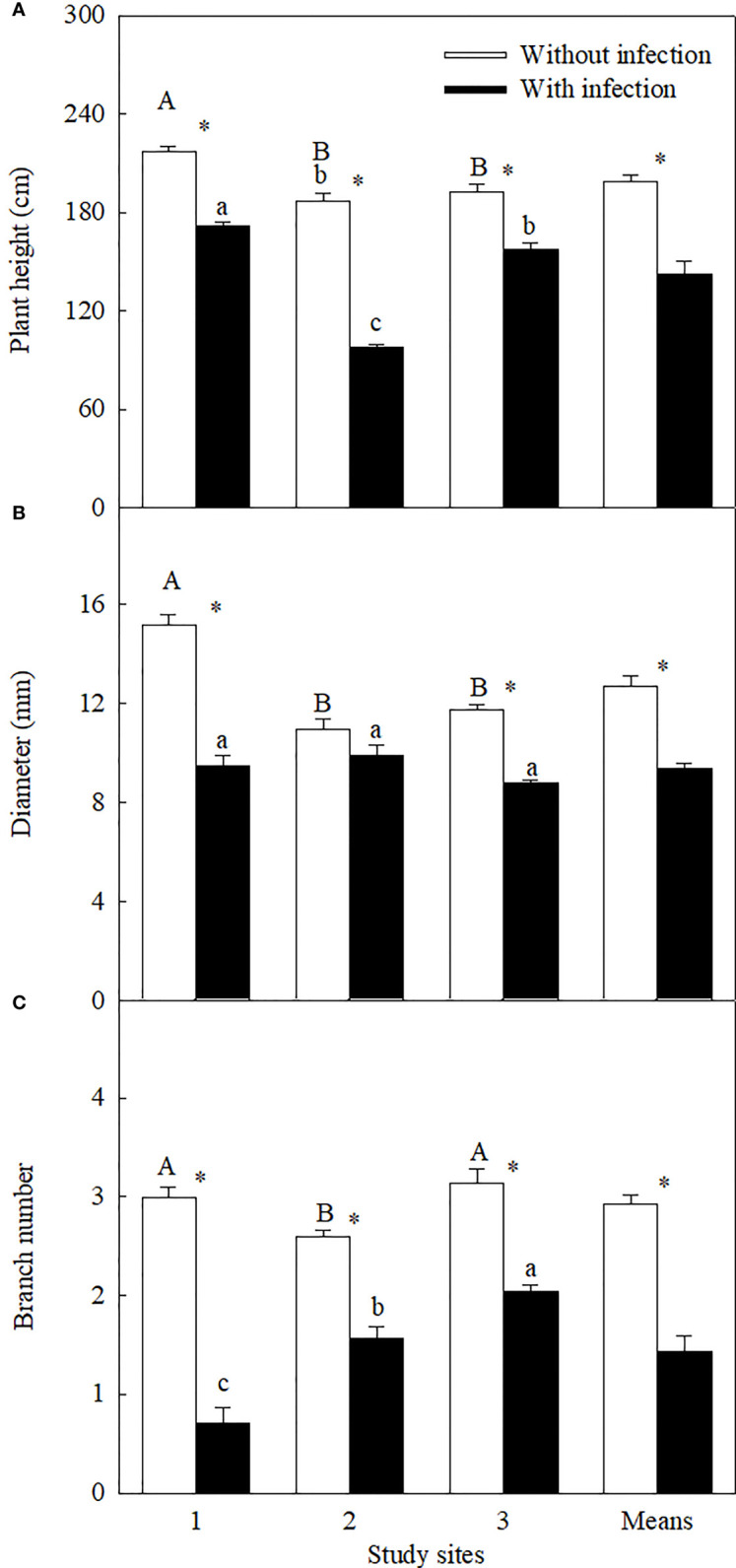
Plant height **(A)**, stem diameter **(B)**, and branch number (log_3_-transformed;) **(C)** of *Ambrosia trifida* uninfected (open bars) and infected (closed bars) by *Cuscuta japonica* at different sites. Means + 1 SE (*n* = 6). Different upper- and lowercase letters indicate significant differences among sites for uninfected and infected plants, respectively (*P* < 0.05; one-way ANOVA); * indicates significant difference between uninfected and infected plants at the same site (*P* < 0.05; independent sample *t*-test).

**Figure 2:**
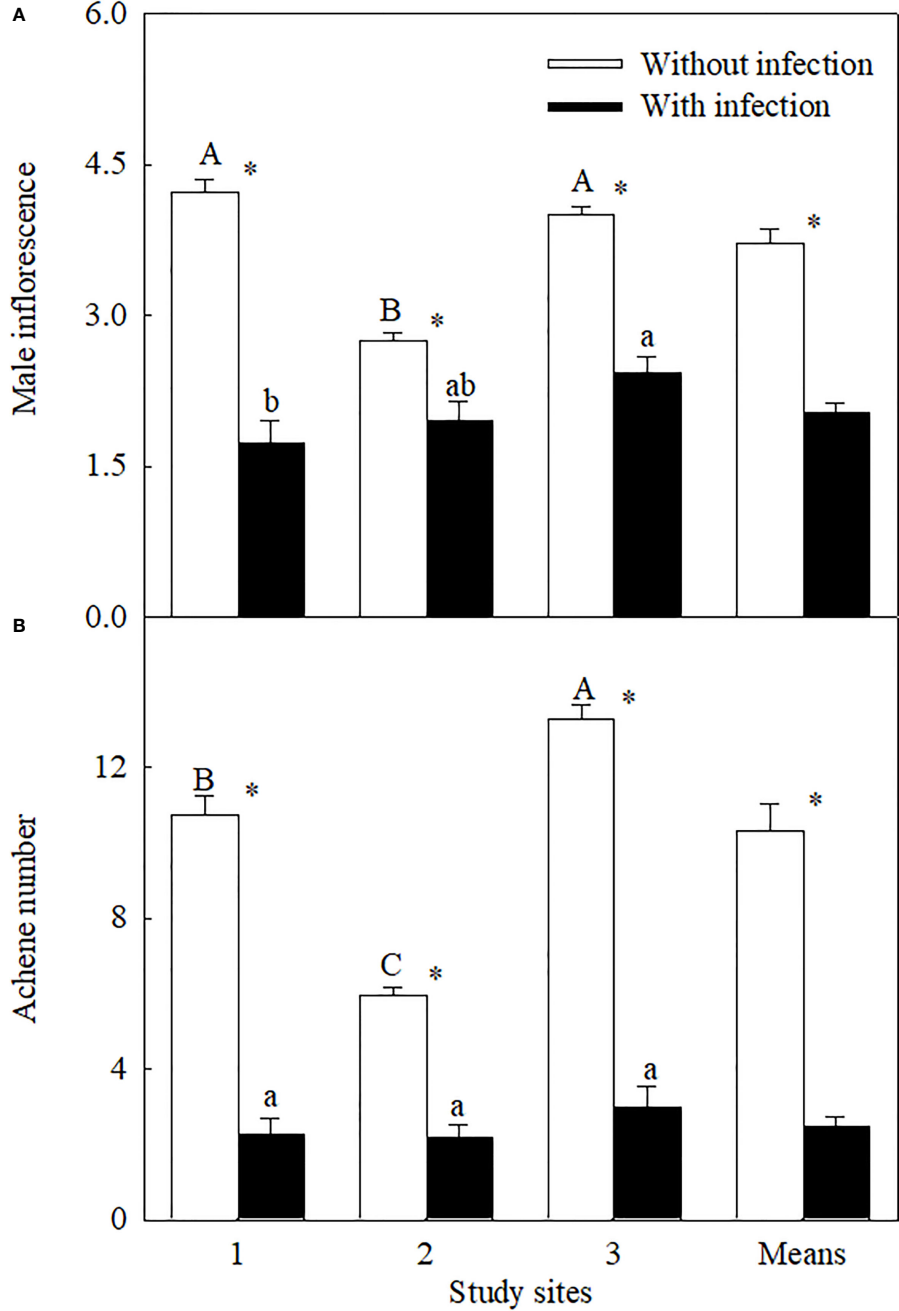
The number of male super-inflorescence (log_3_-transformed;) **(A)** and achene (square-root-transformed; (**B**) of *Ambrosia trifida* uninfected (open bars) and infected (closed bars) by *Cuscuta japonica* at different sites. Means + 1 SE (*n* = 6). Different upper- and lowercase letters indicate significant differences among sites for uninfected and infected plants, respectively (*P* < 0.05; one-way ANOVA); * indicates significant difference between uninfected and infected plants at the same site (*P* < 0.05; independent sample *t*-test).

**Figure 3:**
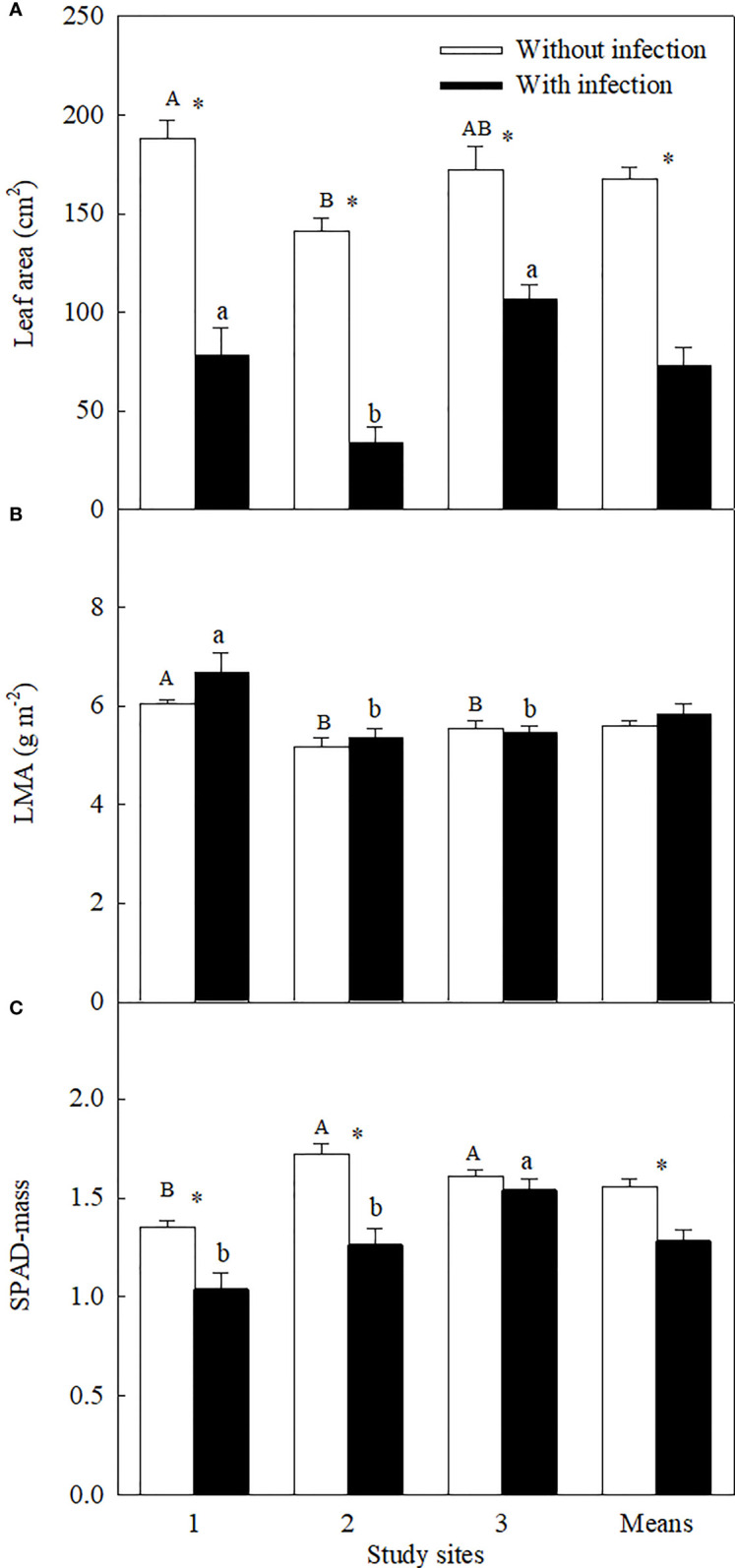
Leaf area **(A)**, leaf dry mass per unit area (LMA, square-root-transformed; **(B)**, and leaf relative chlorophyll content (mass-based SPAD; **(C)** of *Ambrosia trifida* uninfected (open bars) and infected (closed bars) by *Cuscuta japonica* at different sites. Means + 1 SE (*n* = 6). Different upper- and lowercase letters indicate significant differences among sites for uninfected and infected plants, respectively (*P* < 0.05; one-way ANOVA); * indicates significant difference between uninfected and infected plants at the same site (*P* < 0.05; independent sample *t*-test).

Similar levels of parasite infection load on *A. trifida* at different sites, i.e., 45.76 ± 5.72% (site 1), 41.14 ± 8.94% (site 2), and 51.84 ± 7.17% (site 3) (*P* = 0.431), of the main stem length were coiled by the stems of *C. japonica*. The parasite significantly decreased the height, stem diameter, branch number, male super-inflorescence number, achene number, leaf area, and relative chlorophyll content of *A. trifida*, and its effects were significantly different at different sites (**Supplementary Table 3**, **Figures 1**–**3**). On average, the parasite decreased the height, stem diameter, branch number, male super-inflorescence number, achene number, leaf area, and relatively chlorophyll content of the invader by 28.33%, 25.93%, 50.98%, 45.51%, 76.06%, 55.17%, and 17.88%, respectively. Among the seven variables, achene number was the most susceptible to the parasitism, followed by leaf area and branch number, while relative chlorophyll content was the least susceptible followed by stem diameter and plant height.

Among the three sites, stem diameter, branch number, male super-inflorescence number, and achene number were decreased the most by the parasite at site 1, i.e., by 37.32%, 76.22%, 58.99%, and 79.08%, respectively. The decreases in these four variables (except the branch number) were the least at site 2. Plant height (by 47.71%), leaf area (by 76.07%), and relative chlorophyll content (by 26.76%) were decreased the most at site 2.

The LMA of the invader was increased by attack of the parasite at site 1, but not at sites 2 and 3 (**Figure 3B**). The LMA of the invader was bigger at site 1 than at sites 2 and 3, no matter the plants without and with infection.

### C and N concentrations and isotope compositions

**Figure 4:**
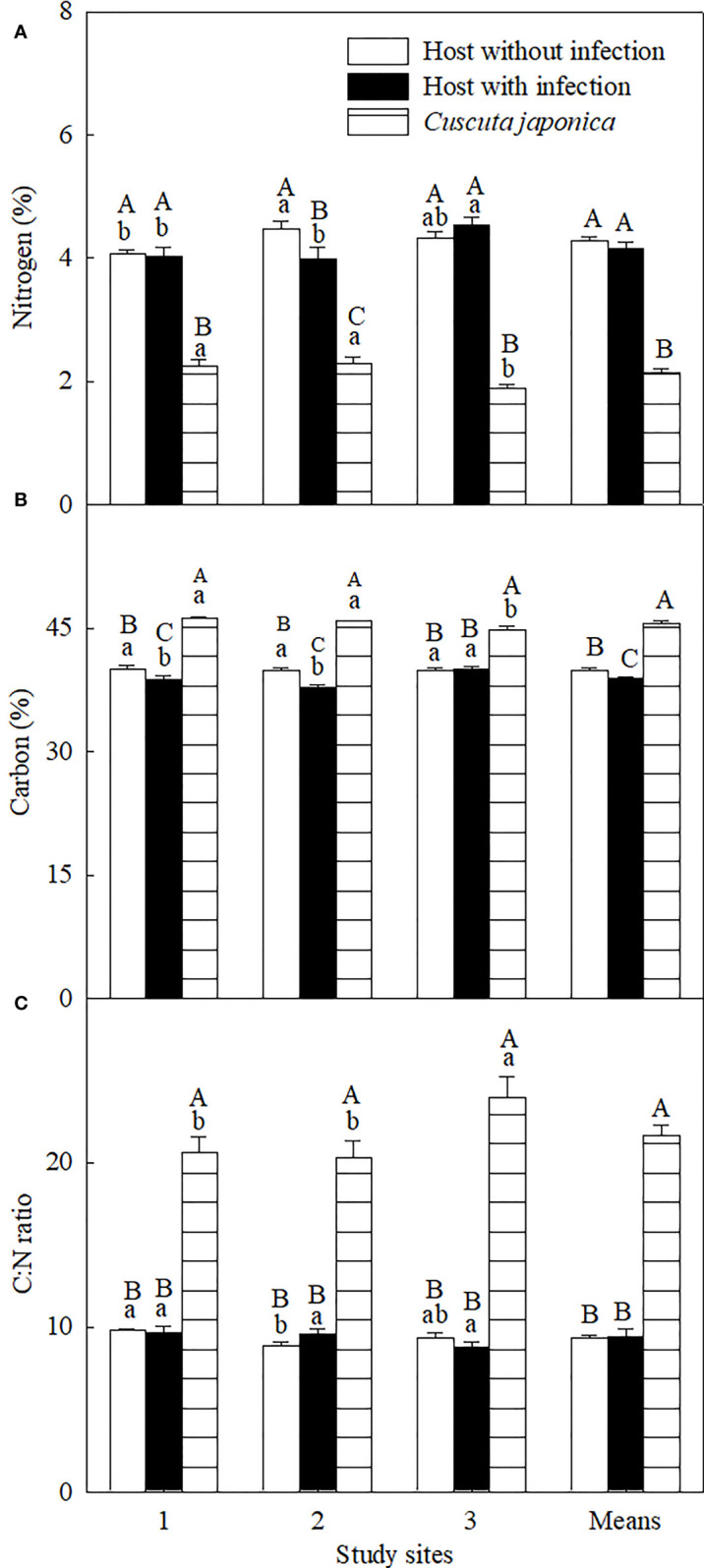
Concentrations of leaf nitrogen **(A)** and carbon **(B)**, and C:N ratio **(C)** of *Ambrosia trifida* uninfected (open bars) and infected (closed bars) by *Cuscuta japonica*, and the stems of *C. japonica* (horizontal-striped bars) at different sites. Means + 1 SE (*n* = 6). Different lowercase letters indicate significant differences among sites for uninfected and infected host plants and the parasite, respectively; different uppercase letters indicate significant differences among uninfected and infected host plants and the parasite at the same site (*P* < 0.05; one-way ANOVA).

Group of species and *C. japonica* treatments and its interaction with study sites (except δ^13^C) significantly influenced all five C- and N-related variables, and sites significantly affected isotope compositions only (**Supplementary Table 4**). On average, the parasite significantly decreased the leaf C concentration by 2.72%, while it did not significantly influence N concentration and C:N ratio (**Figure 4**). Specifically for each site, the parasite decreased leaf N concentration significantly at site 2 but not at sites 1 and 3 and decreased leaf C concentration at sites 1 and 2 but not at site 3, while it did not influence the C:N ratio at any site. The parasite showed a significantly lower stem N concentration but higher C concentration and C:N ratio than its host at any site.

**Figure 5:**
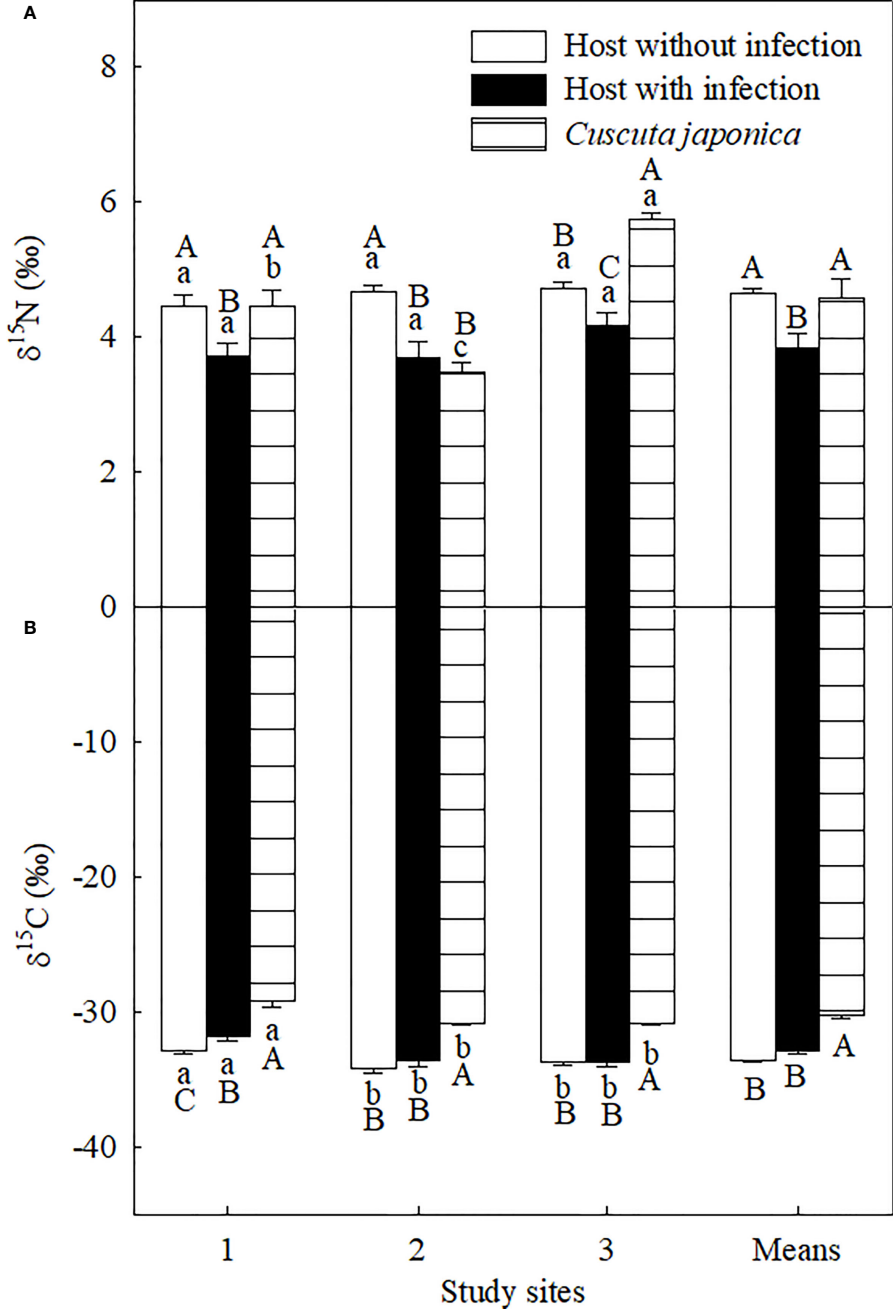
Stable N (δ^15^N; **(A)** and C (δ^13^C; **(B)** isotope compositions of *Ambrosia trifida* uninfected (open bars) and infected (closed bars) by *Cuscuta japonica*, and the stems of *C. japonica* (horizontal-striped bars) at different sites. Means + 1 SE (*n* = 6). Different lowercase letters indicate significant differences among sites for uninfected and infected host plants and the parasite, respectively; different uppercase letters indicate significant differences among uninfected and infected host plants and the parasite at the same site (*P* < 0.05; one-way ANOVA).

The parasite significantly decreased leaf δ^15^N at all three sites and increased leaf δ^13^C at site 1 but not at sites 2 and 3 (**Figure 5**). On average, the parasite significantly decreased leaf δ^15^N by 16.57%, while it did not influence leaf δ^13^C. Compared with its host, the parasite showed significantly higher δ^15^N at sites 1 and 3 but not at site 2, and higher leaf δ^13^C at all study sites. On average, δ^15^N and δ^13^C of the parasite were 8.02% and 18.60% higher than those of its host, respectively.

### Anatomy of the parasitic connection

**Figure 6:**
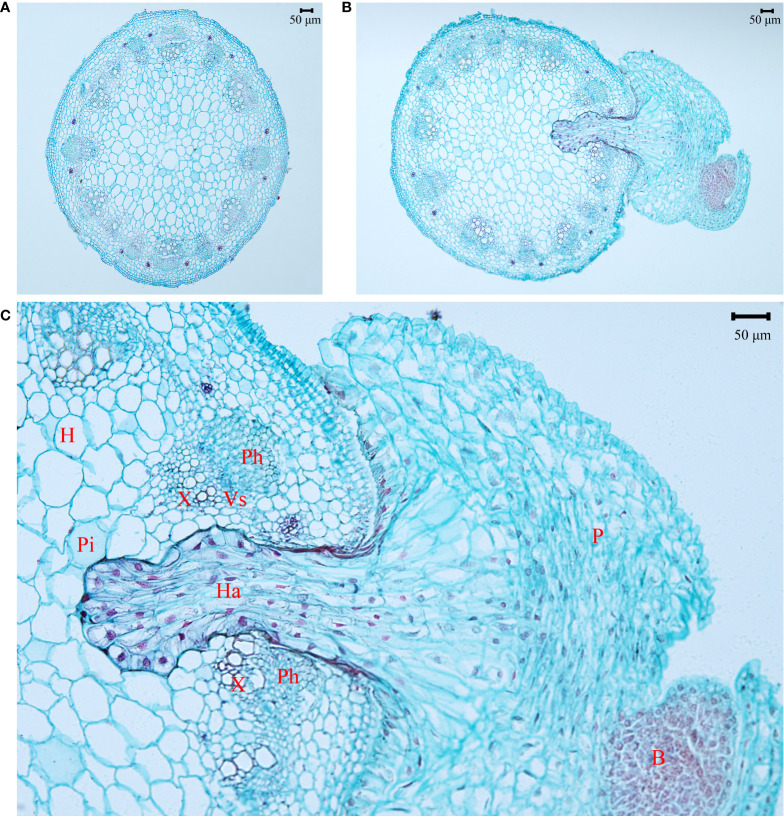
Anatomical structure of *Ambrosia trifida* stems uninfected **(A)** and infected **(B)** by *Cuscuta japonica*, and the magnified area of haustorial endophyte **(C)** in the host stem. Haustorium (Ha) of the parasite (P) penetrated into and reached pith (Pi) of the host stem (H). Ph, phloem; X, xylem; Vs, vascular stripe; B, bud. Bar = 50 μm.

The haustorium of the parasite penetrated into the stem of *A. trifida* (**Figure 6**). The endophyte of the haustorium grew among the parenchyma and vascular bundles of the stem of the invader, including the bundle cap, phloem, xylem, and pith. The apical cells of the haustorium hyphal had conspicuous large nuclei with enlarged nucleoli and dense cytoplasm, showing their active metabolisms.

### Identification of the parasite

The leafless stem of the parasite (1.36 ± 0.16 mm in diameter; *n* = 4 per site, 12 in total) on *A. trifida* was pale yellow at sites 1 and 3 and green at site 2 (with relatively low light), with purplish striations. Its capsules (4.19 ± 0.19 mm long; *n* = 24) were ovoid and had one elongated style with two-lobed stigmas, which reached the middle of the tube. Based on these characteristics, the parasite was preliminarily identified as *Cuscuta japonica* Choisy (Fang et al., 1995), an indigenous parasitic plant in China.

All four rITS2 sequences of the parasite were clustered within a *C. japonica*-dominated clade, including one sequence of *C. europaea* L. (**Supplementary Figure 1A**). All four *trn*Ls sequences of the parasite were clustered within the clade comprising five *trn*Ls sequences *C. japonica* (**Supplementary Figure 1B**). All this evidence proves that the parasite is *C. japonica*.

## Discussion

Our study showed that the parasite significantly suppressed the growth of the invasive plant *A. trifida* in the fields, and its inhibitory effects were greater on reproductive relative to vegetative growth and at high relative to low light habitats. Carbon rather than N constraint may be the mechanism underlying the inhibitory effects of the parasite on the invader. The parasite was identified as *Cuscuta japonica* based on its morphological characteristics and DNA sequences (ITS2 and *trn*L). The phloem-feeding *C. japonica* could also build a haustorial connection with xylem vessels especially at low light habitats and thus directly obtain N from the root, which was confirmed by our stable isotope composition measurements. Many studies found the negative effects of *Cuscuta* species on invasive plants (Qiu et al., 2010; Yu et al., 2011; Yang and Li, 2012; Vrbnicanin et al., 2013; Wang et al., 2019; Wang et al., 2020), but few studies compared their effects on vegetative versus reproductive growth and at different habitats with different resources. Thus, our understanding of the effects of parasitic plants on invasive plants is still limited, preventing the use of the novel agent in control of invasive plants.

### Effect of the parasite on the invader and related mechanisms

Consistent with our hypothesis, both vegetative and reproductive growths of *A. trifida* were severely inhibited by *C. japonica*, which was at least partially associated with its negative effects on leaf area and chlorophyll content. All *A. trifida* plants infected by the parasite were less than 1.72 m tall (**Figure 1A**). For the invader, the decreased height may contribute to a decrease in its growth and ecological impacts. Hejda et al. (2009) found that the invasive plants taller than 1.9 m usually have larger hazard to diversity and evenness of co-occurring native species by forming populations with greater cover and shading the native species. Ferenc and Sheppard (2020) found that plant height is associated with growth, reproduction, and competition by comparing 20 alien annual species. In addition, the decreasing height of *A. trifida* may also affect its pollen dispersal, decreasing its pollination and the risk of causing people’s allergies. The parasite *C. campestris* was also found to significantly decrease the height of *A. trifida* in Serbia (Vrbnicanin et al., 2013) and *Ageratina adenophora* [Spreng.] R.M. King et H. Rob. in China (Qiu et al., 2010). Similarly, *C. australis* can inhibit the growth of invasive plant *Erigeron annuus* [L.] Pers. (Yang and Li, 2012) and *Solidago canadensis* L. (Yang et al., 2015).

Compared with vegetative growth, reproduction of *A. trifida* (numbers of male super-inflorescences and achenes) was more severely inhibited by *C. japonica*. These results were consistent with our previous findings that bur biomass decreased more greatly than aboveground biomass (87.6% to 97.2% vs. 68.7% to 80.7%) for the invasive plant *Xanthium strumarium* L. when infected by *Cuscuta* in the fields (Wang et al., 2019). Strong inhibitory effects of other parasites on reproduction were also found in other invasive plants. For example, the number of burs decreased for *Solanum rostratum* at different sites in northeast China when infected by *C. campestris* (Wang et al., 2020). Prider et al. (2011) found that the native stem hemiparasitic *C. pubescens* is more effective in constraining the reproduction of invasive woody shrub *Cytisus scoparius* than an introduced specialized seed predator *Bruchidius villosus* in South Australia. However, few studies to date have compared the impacts of native parasites on vegetative and reproductive growth of invasive plants.

Competition for C may be one of the reasons that *C. japonica* more strongly inhibited the reproductive relative to vegetative growth of *A. trifida*. Production of super-inflorescence and growth of pollens and fruits are the main C sink for plants at the reproductive growth stage. The phloem-feeding holoparasitic *C. japonica* obtains all carbohydrates exclusively from its host plants. Compared with its host, higher δ^13^C and C concentrations indicated that the parasite is a strong C sink for *A. trifida*. Based on the higher leaf δ^13^C, *A. trifida* plants infected relative to uninfected by *C. japonica* had a more remarkable C constraint. Host plants often initiate defense response to biotic stress when infected by parasites, such as enhancing accumulation of abscisic acid (ABA), reducing stomatal conductance (*g*_s_), and encouraging resource conservation (Ton et al., 2009). A high ABA concentration inhibited leaf expansion (Van Volkenburgh and Davies, 1983) and internode length. Under the stress of parasitic plants, many sensitive invasive hosts increased leaf ABA content (Chen et al., 2011) and reduced *g*_s_ and thus photosynthesis (Frost et al., 1997; Prider et al., 2009; Cirocco et al., 2016), leaf size and stem growth (Shen et al., 2007).

In contrast to the C constraint, infection of *C. japonica* increased the relative availability of N for *A. trifida*, which was shown by the significant decrease in leaf δ^15^N and similar N concentration for the infected and uninfected plants (**Figures 4A**, **5A**). The leaf retention time of N may be increased by the infection, increasing accumulative leaf N-use efficiency. The lower δ^15^N for *A. trifida* plants infected relative to uninfected by *C. japonica* was associated with the higher δ^15^N of the parasite, i.e., more ^15^N was used by the parasite compared with its host. The higher δ^15^N of *C. japonica* was due to the fractionation of ^15^N by roots and leaves of its host.

Stem holoparasitic *Cuscuta* spp. can establish haustorial connections with the phloem and even the xylem vessels of their hosts (**Figure 6**; Hegenauer et al., 2017) and obtain organic N from leaves and/or roots. In most plants, inorganic N assimilation primarily occurred in roots and leaves, and organic N was transported *via* xylem (the major supply route) and phloem, respectively (Kalcsits et al., 2014). Scalon and Wright (2015) found that N is not the limiting nutrient for xylem feeder such as mistletoes.

### Influence of environments on the effects of the parasite

Consistent with the light environments of the study sites, LMA and δ^13^C were higher, while leaf relative chlorophyll content was lower for *A. trifida* at site 1 (with full sunshine) than at sites 2 (shade) and 3 (partial shade). These changes are a typical response to light regimes and could help plants to increase photosynthesis and water use efficiency (Feng et al., 2007; Feng, 2008). For the invader at site 1, the higher decrease in vegetative and reproductive growth caused by *C. japonica* indicates that the inhibitory effects of the parasite are stronger at high relative to low light environments. The higher N and C concentrations of the stems of *C. japonica* at high light environment revealed its strong growth vitality, which may pose severer limitation to the vigor of its host. Similarly, Cirocco et al. (2016) found that the negative effects of *C. pubescens* on growth are stronger for both invasive and native hosts when water availability is high relative to low. These results indicate that the parasite may impose stronger impacts on hosts (particularly reproduction) at more favorable environments.

For the morphological and chemical traits such as height, leaf area, and leaf N and C concentrations, however, the negative impact of *C. japonica* on the invader were stronger at site 2 (shade) than at sites 1 and 3. Intriguingly, the stronger decrease in these traits did not lead to a more significant decrease in vegetative and reproductive growths, which decreased more greatly at sites 1 and 3. Performance of the host depends not only on its production capacity of photosynthate (associated with leaf area and N content) but also on photosynthate allocation between it and the parasite. We hypothesize that more proportion of photosynthate is allocated to the parasite at high light (site 1 and 3) relative to low (site 2) light habitats, posing stronger impacts on the host, which provides a mechanism for the hypothesis that the higher the vigor of the host is, the higher the impact of the parasite pose, as mentioned above. *Cuscuta* spp. may be a stronger C sink than any host organ (Pennings and Callaway, 2002).

The stronger impacts of *C. japonica* on height and leaf area of the invader at site 2 may be associated with its strong water competition with its host. It has been demonstrated that these traits are sensitive to increasing leaf water stress (Koch et al., 2004). Leaf photosynthesis, LMA, root-to-shoot ratio, and mycorrhizal infection rate are generally lower at low light relative to high light habitats (**Figure 3**; Feng et al., 2007; Feng, 2008; Johnson et al., 2015). The impacts of *C. japonica* may further decrease the root-to-shoot ratio and mycorrhizal infection rate (by decreasing photosynthesis and consuming photosynthate), decreasing water as well as nutrient uptake. More importantly, the haustoria of *C. japonica* may more easily penetrate the stems and establish a connection with the xylem of the invader at site 2 compared with other sites (**Figure 6**), where the host is more delicate. This connection made *C. japonica* to intercept the water transported upward by xylem, greatly decreasing water supple for leaf and apical growth points. The same also applies to N transported upward by the xylem, consistent with the lower N concentration in the *A. trifida* infected relative to uninfected by the parasite. This inference was confirmed by our anatomical observation of the haustorial connection and the similar δ^15^N for the parasite and its host at site 2. The leaves of *A. trifida* and the stems of the xylem-feeding *C. japonica* all obtain N from xylem and thus had similar δ^15^N (**Figure 5A**).

## Conclusion

The native *Cuscuta japonica* poses severe C but not N limitation to the invasive plant *Ambrosia trifida* and greatly suppresses its vegetative growth and reproduction performance in the fields. The negative impacts of the parasite are stronger at high relative to low light habitat, supporting the hypothesis that the higher the vigor of the host is, the higher the impact of the parasite pose. The significant decrease in plant height and branch number and the approximate failure in achene production indicate that the native *Cuscuta* may greatly decrease the ecological impacts of the invader on recipient ecosystems in the current and even the following growth season. These results indicate, for the first time, that the parasite is a promising biological agent for the noxious invader in China. A better understanding of the mechanisms underlying the impacts of native parasites on invasive plants in terms of physiology and ecology will shed light on the potential use of the parasites in controlling noxious invasive weeds, and thus the parasites’ impacts on invasive plants merit further study, through either controlled experiments or larger-scale field studies.

## Data availability statement

The datasets presented in this study can be found in online repositories. The names of the repository/repositories and accession number(s) can be found in the article/**Supplementary Material**.

## Author contributions

W-BW and Y-LF conceived and designed the experiment. W-BW, F-FG, W-WF, and Q-YW performed the experiment and analyzed the data. W-BW and Y-LF interpreted the analyses and wrote the manuscript. All authors have read and approved the submitted manuscript.

## Funding

This work is financially supported by the National Natural Science Foundation of China (32171667, 31971557 and 32171666) and the National Key Research and Development Program of China (2017YFC-1200101).

## Acknowledgments

The authors are grateful to Dr. Ming-Chao Liu for assistance in sampling in the fields in 2018 and 2019, Jin-Yi Xu for assistance in measuring the concentrations and stable isotope compositions of C and N, and three reviewers for their helpful and constructive comments.

## Conflict of interest

The authors declare that the research was conducted in the absence of any commercial or financial relationships that could be construed as a potential conflict of interest.

## Publisher’s note

All claims expressed in this article are solely those of the authors and do not necessarily represent those of their affiliated organizations, or those of the publisher, the editors and the reviewers. Any product that may be evaluated in this article, or claim that may be made by its manufacturer, is not guaranteed or endorsed by the publisher.
