# Supplementary material for: The native stem holoparasitic Cuscuta japonica suppresses the invasive plant Ambrosia trifida and related mechanisms in different light conditions in northeast China
Source: Front Plant Sci. 2022 Sep 23;13:904326. doi: 10.3389/fpls.2022.904326 (PMC9539100; doi:10.3389/fpls.2022.904326)
Supplement: Supplementary file 1 [file DataSheet_1.docx]

**Supplementary Table 1**

Information on the sampling sits of the stems of the *Cuscuta* in Shenyang, Liaoning Province, northeast China.

Year Sites Coordinates Altitude (m)

2018 North bank, Hunhe river N 41°48'36.51", E 123°33'21.32" 50

2018 Benchland, Hunhe river N 41°48'04.20", E 123°33'42.88" 45

2019 West slope, Shenshui East N 41°48'50.75", E 123°33'04.98" 54

2020 East slope, Shenshui East N 41°48'54.85", E 123°33'07.28" 59

**Supplementary Table 2**

Information on the *Cuscuta* species whose consequences of ITS2 (A) and *trn*L (B) were used to construct phylogenetic trees.

**Supplementary Table 2A**

**Species Host Organ Sample code Country People and year* Accession No.**

*Cuscuta monogyna*

(outgroup) -- -- 654028120703003 -- Li and Fan (2013) KF454373

*C. japonica* *Ambrosia trifida* stem *Ambrosia trifida* L China (Shenyang) Wang et al. (2018) MK070163

*C. japonica A. trifida* stem 2019-335S.2.2 China (Shenyang) Wang and Gao (2021) MW486627

*C. japonica* *A. trifida* stem 2020-391S.14-2.2 China (Shenyang) Wang and Gao (2021) MW486637

*C. japonica* *A. trifida* stem 2020-390S.15-2.2 China (Shenyang) Wang and Gao (2021) MW486639

1. *C. japonica* *Panax ginseng*  -- DH1 China (Jilin) Chen et al. (2012) JF431541
2. *C. japonica* -- -- KIOM201701018784 clone 2 -- Park (2020) MT984391
3. *C. japonica* -- -- KIOM201701018784 clone 1 -- Park (2020) MT984390
4. *C. japonica* -- -- Q817 China (Qinling) Xu et al. (2018) MH711640
5. *C. japonica* *Rubus idaeus* -- DH13 China Gao et al. (2013) KC542379
6. *C. europaea* -- -- SunH-07ZX-3211 China (Qinling) Xu et al. (2020) MH809152
7. *C. japonica* -- -- Ketchersid and Heintze s.n. USA (Texas) García and Martín (2007) DQ924571
8. *C. japonica* *Dictamnus dasycarpus* -- SN141020 China (Fushun) Yuan et al. (2015) KP015829
9. *C. japonica* -- -- # USA (Pennsylvania) McNeal et al. (2007) EU330320
10. *C. japonica* -- -- CY-C-JAPONIC 006 -- Cheng (2005) DQ211588
11. *C. japonica* -- -- Ge130851 China (Qinling) Xu et al. (2018) MH710706
12. *C. lupuliformis* -- -- (PAC) JRM03.0808 USA (Pennsylvania) McNeal et al. (2007) EU330321
13. *C. reflexa*  -- -- # USA (Pennsylvania) McNeal et al. (2007) EU330322

* indicates the people who deposited the sequence in this year. The first four *C. japonica* sequences are from the present study.

**Supplementary Table 2B**

**Species Host Organ Sample code Country People and year* Accession No.**

*Cuscuta exaltata*

(outgroup) -- -- -- USA (Pennsylvania) McNeal et al. (2007) EU189132

*C. japonica* *Ambrosia trifida* stem 2019-335S.1.2 China (Shenyang) Wang and Gao (2021) MW618665

*C. japonica* *A. trifida* stem 2020-391S.14-1.2 China (Shenyang) Wang and Gao (2021) MW618675

*C. japonica* *A. trifida* stem 2020-390S.15-1.3 China (Shenyang) Wang and Gao (2021) MW618676

*C. japonica* *A. trifida* stem 3.1(2018) China (Shenyang) Wang et al. 2019 MK879389

1. *C. japonica* -- -- 853 Japan (Hashimoto) Stefanovićet et al. (2004) AY101170
2. *C. japonica* -- -- KIOM-2016-279 -- Park and Moon (2018) MH780080
3. *C. japonica* -- -- Ketchersid and Heintze s.n. USA (Texas) García and Martín (2007) EF152064
4. *C. japonica* -- -- -- -- Hsieh et al. (2006) DQ852345
5. *C. japonica* -- -- -- -- Hsieh et al. (2006) DQ852344
6. *C. lupuliformis*  -- -- MA-94616 Germany García and Martín (2007) AJ428058
7. *C. reflexa Pelargonium zonale* stem -- Germany (Kiel) Funk et al. (2007) AM711640

* indicates the people who deposited the sequence in this year. The first four *C. japonica* sequences are from the present study.

**Supplementary Table 3**

Two-way ANOVA showing effects of *Cuscuta japonica* treatments (C; *n* = 2), study sites (S; *n* = 3) and their interaction on plant height (cm), stem diameter (mm) at 40 cm height, branch number, male inflorescence number (Inflor.), achene number, leaf area (LA), leaf mass per area (LMA) and relative chlorophyll content (SPAD) of *Ambrosia trifida*.

Height Diameter Branch Inflor. Achene LA LMA SPAD

C **< 0.0001 < 0.0001 < 0.0001 < 0.0001 < 0.0001 <0.001** 0.182 **< 0.0001**

327 130 225 190 471 107 1.87 34.8

30231 101 21.8 25.2 543 72480 0.514 0.775

S **< 0.0001 < 0.0001 < 0.0001 < 0.0001 < 0.0001** <**0.001** <**0.001 < 0.0001**

92.1 22.0 20.7 18.9 45.9 12.6 14.4 22.8

17023 34.3 3.83 5.02 106 17032 7.92 1.02

C × S **< 0.0001 < 0.0001 < 0.0001 < 0.0001 < 0.0001** 0.091 0.249 **0.008**

28.8 21.2 17.1 17.0 31.2 2.60 1.46 5.59

5325 33.2 3.15 4.52 71.9 3512 0.802 0.249

Note: The 1^st^, 2^nd^, and 3^rd^ row for each factor indicate *P*-value*, F*-value, and the sum of square, respectively. Male inflorescence number and branch number are Log_3_-transformed, and achene number and LMA are square-root transformed. Significant effects (*P* < 0.05) are shown in bold.

**Supplementary Table 4**

Two-way ANOVA showing effects of the groups of species and *Cuscuta japonica* treatments (G; *n* = 3), study sites (S; *n* = 3) and their interaction on tissue carbon (C) and nitrogen (N) concentrations, their ratio (C:N) and stable isotope compositions (δ^13^C and δ^15^N) of the parasite and *Ambrosia trifida*.

N C C:N δ^15^N δ^13^C

G **< 0.0001** **< 0.0001** **< 0.0001** **< 0.0001** **< 0.0001**

288 286 446 17.5 109

55.2 498 1888 7.04 120

S 0.324 0.165 0.067 **< 0.0001 < 0.0001**

1.15 1.87 2.84 21.3 28.4

0.220 3.25 12.0 8.57 31.4

G × S **< 0.001** **0.001** **0.001** **< 0.0001** 0.384

5.54 5.43 5.43 10.7 1.06

2.12 18.9 45.9 8.61 2.35

Note: The 1^st^, 2^nd^, and 3^rd^ row for each factor indicate *P*-value*, F*-value, and the sum of square, respectively. Significant effects (*P* < 0.05) are shown in bold.
