# Supplementary figures and images for: The native stem holoparasitic Cuscuta japonica suppresses the invasive plant Ambrosia trifida and related mechanisms in different light conditions in northeast China
Source: Front Plant Sci. 2022 Sep 23;13:904326. doi: 10.3389/fpls.2022.904326 (PMC9539100; doi:10.3389/fpls.2022.904326)

A

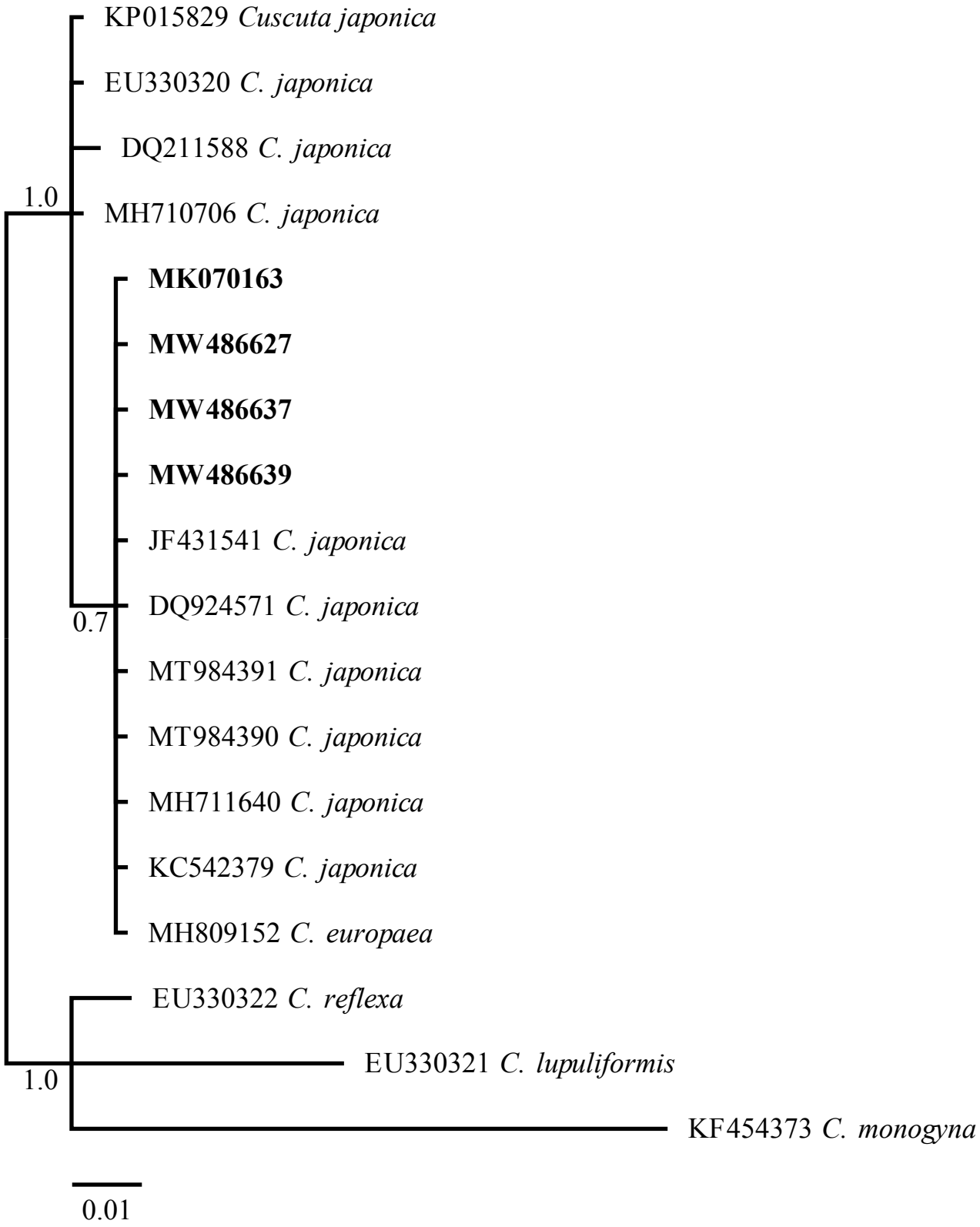

B

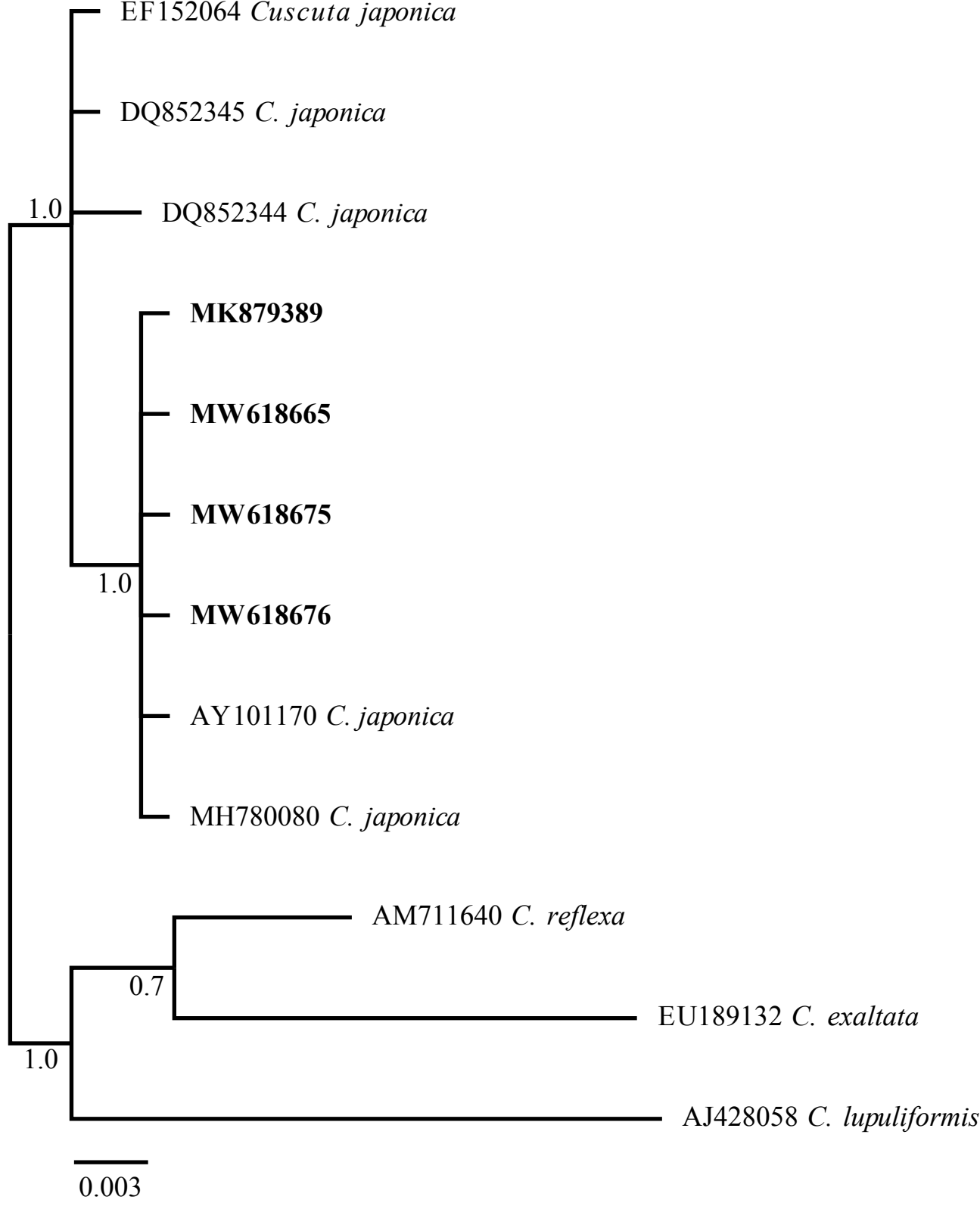

Supplement: Supplementary file 2 [file DataSheet_1.pdf]
